# Partial Fibular Head Osteotomy is an Alternative Option in Treatment of Posterolateral Tibial Plateau Fractures: A Retrospective Analysis

**DOI:** 10.3389/fsurg.2022.915814

**Published:** 2022-05-09

**Authors:** Yao Lu, Gen Wang, Cheng Ren, Yibo Xu, Qiang Huang, Shan Fan, Ming Li, Kun Zhang, Zhong Li, Qian Wang, Teng Ma

**Affiliations:** ^1^ Department of Orthopaedic Surgery, Honghui Hospital, Xi’an Jiaotong University, Xi'an, Shaanxi, China; ^2^ Bioinspired Engineering and Biomechanics Center (BEBC), The Key Laboratory of Biomedical Information Engineering of Ministry of Education, School of Life Science and Technology, Xi’an Jiaotong University, Xi’an, China; ^3^ Orthopaedics Institute of Chinese PLA, 80th Hospital, Weifang, China

**Keywords:** short-term effects, retrospective analysis, fracture, tibial plateau, osteotomy

## Abstract

**Objective:**

This study aimed to evaluate the short-term effects of partial fibular head osteotomy for treating posterolateral tibial plateau fractures.

**Methods:**

A retrospective analysis was performed on 25 patients with posterolateral tibial plateau fractures who were treated using a partial fibular head osteotomy approach. Computed tomography was performed for fracture typing and evaluation. The mode of injury, time from injury to surgery, time for fracture union, range of motion of the knee, and complications were recorded. Knee joint function was evaluated using the Hospital for Special Surgery Mayo Score (HSS).

**Results:**

The mean follow-up period was 21.5 (range, 12–36) months. Fracture united in all patients and the average clinical healing time for fractures was 11.2 ± 1.9 (range, 8–16) weeks. The mean time from injury to surgery was 3.1 ± 1.8 (range, 1–10) days. The mean range of flexion was 131.6° ± 12.5° (range, 110°–145°). The mean range of extension was 1.4°–4.2° (range, −5°–10°). The mean HSS at the final follow-up was 93.5 ± 5.4 (range, 79–100). None of the patients exhibited symptoms of common peroneal nerve injury, knee instability, or upper tibiofibular joint injury. One patient had a superficial infection and was treated with surgical dressing.

**Conclusion:**

The partial fibular head osteotomy approach is a feasible alternative for treating posterolateral tibial plateau fractures.

## Introduction

Posterolateral tibial plateau fractures remain a great challenge for orthopedic surgeons (1). Isolated posterolateral tibial plateau fractures are rare and often part of complex tibial platform fractures (2). For intra-articular fractures, anatomic reconstruction of the articular surface and fixation for displaced posterolateral fractures of the tibial plateau are important for knee joint range of motion and stability (3, 4). A variety of approaches have been used for open reduction and internal fixation of posterolateral tibial plateau fractures (3, 5–7). The standard anterolateral approach can be used to visualize the articular surface and manage lateral fracture fragments of the lateral tibial plateau. However, this approach has limited exposure for fractures involving the posterolateral articular surface and does not permit the insertion of posterior fixation (8).

The posterolateral approach can visualize the posterolateral articular surface, manage posterolateral fracture fragments, and provide posterior implants for effective fixation (9). However, this approach usually causes more soft tissue damage, such as knee posterolateral ligament complex injury, common peroneal nerve injury, and inferolateral geniculate artery injury (10). The transfibular approach (fibular head osteotomy or fibular neck osteotomy) can intuitively expose posterolateral articular injuries. Some studies have reported that the transfibular approach has no adverse effects (3, 11), while others reported some disadvantages, such as common peroneal nerve injury, fibular nonunion, and destabilization of the proximal tibiofibular joint (10).

**Figure 1:**
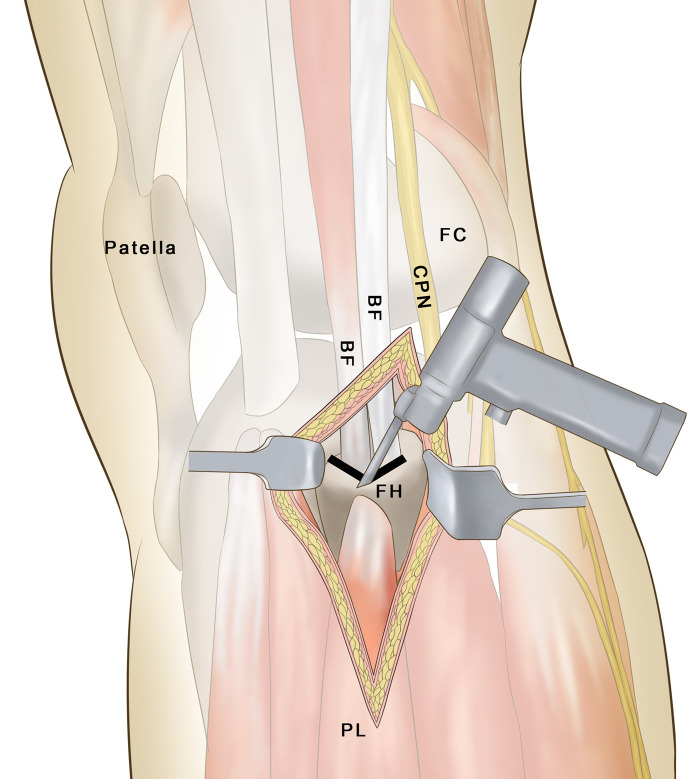
A schematic of the V-shaped osteotomy. FH, fibular head; CPN, common peroneal nerve; BF, biceps femoris; PL, peroneus longus; FC, femoral condyle.

Therefore, a modified transfibular approach, called ‘partial fibular head osteotomy’ (Figure 1), was developed to manage posterolateral tibial plateau fractures. In this study, the benefits and limitations of the partial fibular head osteotomy approach in treating posterolateral tibial plateau fractures were evaluated.

## Methods

### Inclusion and Exclusion Criteria

The inclusion criteria were as follows: (i) diagnosis of fresh closed tibial plateau fractures with normal knee joint movement before injury; (ii) partial fibular head osteotomy approach for the treatment of posterolateral tibial plateau fractures; (iii) postoperative follow-up of ≥12 months; (iv) no serious vascular or nerve injury; and (v) retrospective study. The exclusion criteria were as follows: (i) pathological fracture; (ii) severe osteoarthritis and osteoporosis; (iii) patients who could not be contacted; and (iv) those with incomplete clinical data before and after surgery.

### Patient Information

A retrospective study reviewed a consecutive series of 33 patients with tibial plateau fractures who presented at the authors’ center between January 2017 and January 2020. Eight patients were excluded according to the aforementioned criteria, including two with osteoarthritis, two lost to follow-up, and four with incomplete clinical data before or after surgery. Finally, 25 patients were enrolled in the present study.

**Figure 2:**
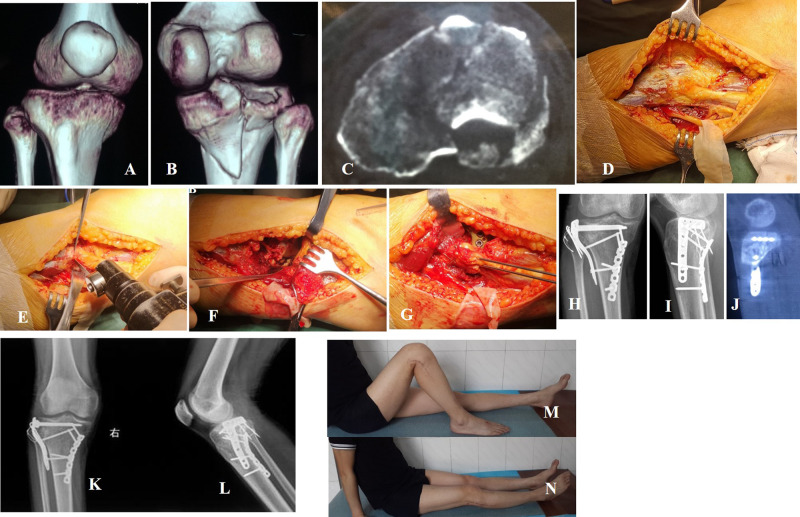
A 26-year-old male patient suffered a posterolateral fracture (Schatzker V) caused by falling from a height. (**A**–**C**) Preoperative CT scan and reconstruction confirmed fracture type and obvious displacement. (**D**) The common peroneal nerve was carefully identified and protected. (**E**) ‘V’ shape osteotomy of the partial fibular head. (**F**) Posterolateral articular surface under direct observation. (**G**) The fibular osteotomy was fixed with a K-wire tension band. (**H**–**J**) X-ray and CT scans show an anatomical reduction, good internal fixation, and recovery of the smooth articular surface. (**K**,**L**) The fracture healed with no internal fixation failure 1 year after surgery. (**M**,**N**) Knee function was excellent at 1 year after surgery.

The preoperative examination included anteroposterior and lateral radiographs of the knee joint, two-dimensional computed tomography (CT) scanning, and three-dimensional reconstruction of the proximal tibial joint (Figures 2A–C). Patient demographic characteristics, including age, sex, mechanism of injury, side of injury, time from injury to surgery, and type of fracture (Dubberley Schatzker and 4 column classification system (12, 13), are shown in Table 1. The study was reviewed and approved by the Ethics Committee of Honghui Hospital, Xi’an Jiaotong University. All patients provided written and signed informed consent. All methods were performed in accordance with the relevant guidelines and regulations.

**Table 1:** Summary of the patiant variables and outcomes of posterolateral tibial plateau fractures.

Patient No.	Age (years)	Sex (M/F)	Side (L/R)	Mode of injury	Four columns Classification	Schatzker Classification	Time from injury to surgery (d)	Time for fracture union (weeks)	follow-up (month)	Extension (°)	Flexion (°)	HSS score
1	51	F	L	Fall	PL	I	2	8	36	0	145	100
2	60	M	L	Fall	PL	III	3	12	12	5	135	97
3	40	M	L	Fall	PL + AL	II	2	13	24	5	140	95
4	26	M	R	Falls from a height	PL + PM	V	3	16	36	−5	135	93
5	65	F	L	Fall	PL	III	2	14	16	0	140	96
6	50	F	R	Fall	PL + AL	II	3	11	20	0	145	100
7	36	F	L	Traffic accident	PL + AL	II	5	12	18	10	135	90
8	36	F	L	Traffic accident	PL + AL	I	3	13	36	0	110	94
9	53	M	R	Traffic accident	PL	II	2	10	16	0	125	88
10	55	F	L	Fall	PL + AL	II	6	8	12	0	120	95
11	40	F	L	Fall	PL + PM	V	3	10	24	0	140	98
12	42	M	R	Falls from a height	PL + AL	II	1	12	36	0	145	98
13	46	M	L	Traffic accident	PL + AL	II	2	12	12	0	115	79
14	51	F	R	Fall	PL + AL + PM	V	3	10	24	10	120	90
15	63	F	L	Traffic accident	PL + AL + PM	V	3	12	18	5	140	97
16	33	M	R	Traffic accident	PL + AL	II	4	11	32	0	130	93
17	52	F	L	Fall	PL	II	3	8	16	−5	140	95
18	39	F	L	Fall	PL + AL + PM + PL	V	10	12	14	5	125	81
19	31	F	L	Traffic accident	PL + PM	V	3	10	20	0	100	94
20	52	M	R	Fall	PL	III	3	8	20	0	145	96
21	41	M	R	Falls from a height	PL + AL + PM + PL	V	3	13	32	−5	115	86
22	58	F	R	Traffic accident	PL + AL	II	2	12	24	0	140	98
23	64	F	L	Traffic accident	PL + PM	V	2	10	13	10	130	92
24	22	M	R	Fall	PL + AL	II	2	12	16	0	130	98
25	61	M	L	Fall	PL	II	3	11	12	0	145	95

*M, male; F, female; L, left; R, right; AL, anterolateral; PL, posterolateral; AM, anteromedial; PM, posteromedial; HSS, The Hospital for Special Surgery Score.*

### Surgery

Two senior doctors completed the operations on all the patients. General anesthesia or epidural anesthesia combined with subarachnoid block anesthesia was administered, and the patients were placed in the supine position. A balloon tourniquet with a pressure of 50 kPa was used on the lower extremities.

The skin incision longitudinally followed the fibula, with the proximal end 8–10 cm above the plane of the fibula head extending 10–15 cm along the fibula to the distal end. The common peroneal nerve was carefully identified and protected (Figure 2D).

A V-shaped osteotomy was performed on the margin of the stop point of the biceps femoris tendon on the fibula head (Figures 1, 2E). The height of the osteotomy was defined as the height of the upper tibiofibular notch of the proximal lateral periarticular plate of the tibia. The pendulum saw was cut to the lateral cortex, and the contralateral cortex was slowly truncated using an osteotome. The lateral and posterolateral parts of the tibial plateau were fully exposed (Figure 2F). Following reduction, the subchondral bone was temporarily fixed from the outside to the inside using Kirsch wires. The reduction was confirmed visually and radiographically. The lateral locking plate of the proximal tibia (Tianjin Zhengtian Medical Instrument Co., Ltd., Tianjin, China) was placed between the posterolateral tibial plateau and the fibular head. The fibular osteotomy was fixed with a K-wire tension band (Figure 2G).

### Postoperative Treatment and Efficacy Evaluation

Antibiotics were administered 30 min before surgery and 24–48 h after surgery. On the second day after surgery, radiography (Figures 2H,I) and CT scan (Figure 2J) were performed to observe fracture reduction and fixation. Each participant used a continuous-passive-motion machine. Following the passive activity regimen, patients were encouraged to undertake active, non-weight-bearing knee flexion and extension activities. Patients were followed up at four, eight, and 12 weeks after surgery. In most patients, fractures healed by 12 weeks post-surgery, based on X-ray scan examination. At this point, the use of crutches was gradually phased out to promote weight-bearing walking. Thereafter, the patients were followed-up every three months, which included assessments of the range of active and passive motion, stability, and knee joint fracture healing based on AP and lateral X-ray examination (plus CT scan if necessary), and any complications arising during the follow-up period. Knee joint function was assessed by the Hospital for Special Surgery Mayo Score (HSS) knee joint clinical function scoring system (14), and HSS scores were graded as excellent (85), good (70–84), fair (60–69), and poor (59).

## Results

Twenty-five patients were included in the study, with an average age of 46.7 (range, 22–65) years. All fractures were closed. According to the Schatzker classification system, there were two patients with type I fractures, eleven patients with type II, two patients with type III, and eight patients with type V, respectively. The mean time from injury to surgery was 3.1 ± 1.8 (range, 1–10) days (Table 1). All patients were followed up for 12–36 months [mean (21.5 ± 8.5) months]. All fractures had united, and the average clinical healing time for fractures was 11.2 ± 1.9 (range, 8–16) weeks (Figures 2K,L). The mean range of flexion was 131.6° ± 12.5° (range, 110°–145°) (Figure 2M). The mean range of extension was 0° (range, −5°–10°) (Table 1) (Figure 2N). The mean HSS at the final follow-up was 93.5 ± 5.4 (range, 79–100). Based on the HSS, 23 (92%) patients had excellent results, and two (8%) had good results (Table 1). None of the patients exhibited symptoms of common peroneal nerve injury, knee instability, or upper tibiofibular joint injury. One patient had a superficial infection and was treated with surgical dressing.

## Discussion

Posterolateral tibial plateau fractures account for 15% of all tibial plateau fractures, with isolated posterolateral fractures occurring at approximately 7% (15). For intra-articular fractures, anatomic reconstruction of the articular surface and fixation for displaced posterolateral fractures of the tibial plateau are important for knee joint range of motion and stability. To expose the posterolateral articular surface, the proximity of the common peroneal nerve and the posterolateral ligamentous structures is a great challenge. This leads to multiple surgical approaches to access the region and improve visibility (10). For most tibial plateau fractures, the traditional anterolateral approach is familiar and safe for orthopedic surgeons. However, it fails to visualize the articular surface of the posterolateral tibial plateau and applies it to the buttress plate (16). Carlson (17) reported that posterolateral fracture fragments can be directly exposed and performed using a posterolateral approach with a buttress plate for fixation; however, it also presents some disadvantages, including the risk of injury to the anterior tibial artery, inferolateral geniculate artery, and common peroneal nerve (10). Sun et al. used a posteromedial inverted L-shaped approach to treat posterolateral tibial plateau fractures (18). This sometimes necessitates cutting the medial head of the gastrocnemius to achieve good reduction and fixation for posterolateral fracture of the simple split, and its use has not been reported for the treatment of posterior lateral depression fractures. Frosch et al. reported an anterolateral and posterolateral arthrotomy approach, which can clearly visualize the joint surface (19). However, its disadvantages include risk to surrounding soft tissue and the common peroneal nerve.

The transfibular approach is an advantageous choice for managing posterolateral fracture of the tibial plateau, especially for some complex posterolateral fractures and some posterolateral fractures combined with a proximal fibula fracture. The fibular head osteotomy approach, described by Yu et al., can provide a good view of the reduction and internal fixation to treat posterolateral tibial plateau fractures (11). Chen et al. reported that the fibular neck osteotomy approach in treating posterolateral tibial plateau fractures can provide excellent visualization of posterolateral articular injury and has no adverse effects (3). Previous studies showed that the fibular osteotomy approach is advantageous for exposing posterolateral fractures (3, 11). However, there is a significant risk of injury to the common peroneal nerve due to the location of the plate between the upper tibia-fibula joint and the lower osteotomy position. Although the aforementioned studies reported good results with the use of the transfibular approach, there is greater limited utility due to the destabilization of the proximal tibiofibular joint and the common peroneal nerve injury (20). Therefore, a partial fibular head osteotomy approach was designed. This approach is distinguished by careful evaluation of the range of the biceps tendon on the fibular head and performing a ‘V’-shaped osteotomy at the edge of the connecting point after exposing the common peroneal nerve (Figure 2E). The osteotomy height matched that of the lateral periarticular bone plate of the fibular proximal end of the fibular notch of the tibia. This may prevent instability of the proximal tibiofibular joint. The fibular head was turned to the proximal end, the capsule ligament and coronary ligament were cut off, and the lateral meniscus was sutured to the proximal end to fully expose the lateral and posterior aspects of the tibial plateau. In most cases, the plate occupied part of the original position of the fibular head, a portion of the tibia-fibular notch was removed to ensure smoothness of the lateral side of the fibular head and avoid causing damage to the common peroneal nerve. In the present study, none of the patients experienced complications, such as common peroneal nerve palsies, pain in the lateral knee, nonunion at the osteotomy site, or knee instability. One patient had a superficial infection and was treated with surgical dressing.

The advantages of the fibular neck osteotomy approach include (1) no vital vessel structure; (2) avoiding the common peroneal nerve and extensive soft tissue injury; (3) the ‘V’-shaped osteotomy of the fibular head reduces sagittal displacement risk while increasing contact area, which benefits healing; (4) the height of the ‘V’ shape osteotomy matching the height of the lateral joint plate of the proximal tibia on the tibiofibular notch and the biceps femoris insertion point lowers the risk of damaging the superior tibiofibular joint; (5) partial removal of the articular surface from the tibiofibular notch on the fibular head, avoiding stepwise stimulation and consequent nerve damage; and (6) articular surface areas can be reset under direct observation. The disadvantages of this method include (1) fibular iatrogenic fractures during exposure of the tibial plateau and lateral plate placement occupying part of the superior tibiofibular joint; (2) difficulty in removing fixation materials at a late stage; and (3) exceptional cases requiring removal of fixation materials necessitating the destruction of some lateral structures.

This study was limited by the small number of cases (*n* = 25), some bias in patient selection, short follow-up period, and lack of biomechanical studies. Furthermore, prospective studies with a greater number of patients and long-term follow-up are needed to determine the safety and effectiveness of this approach.

## Conclusion

The partial fibular head osteotomy approach can fully expose the posterolateral tibial plateau fractures and provide stable internal fixation of these fractures. Therefore, this approach may provide a feasible alternative for treating posterolateral tibial plateau fractures.

## Data Availability

The original contributions presented in the study are included in the article/Supplementary Material, further inquiries can be directed to the corresponding author/s.
